# Reply: Does the polyubiquitination pathway operate inside intact chloroplasts to remove proteins?

**DOI:** 10.1093/plcell/koae105

**Published:** 2024-05-13

**Authors:** R Paul Jarvis, Jialong Li, Rongcheng Lin, Qihua Ling, Yuping Lyu, Yi Sun, Zujie Yao

**Affiliations:** Section of Molecular Plant Biology, Department of Biology, University of Oxford, Oxford OX1 3RB, UK; Key Laboratory of Photobiology, Institute of Botany, Chinese Academy of Sciences, Beijing, 100093, China; Key Laboratory of Photobiology, Institute of Botany, Chinese Academy of Sciences, Beijing, 100093, China; National Key Laboratory of Plant Molecular Genetics, CAS Centre for Excellence in Molecular Plant Sciences, Institute of Plant Physiology and Ecology, Chinese Academy of Sciences, Shanghai, 200032, China; CAS-JIC Center of Excellence for Plant and Microbial Sciences (CEPAMS), Institute of Plant Physiology and Ecology, Chinese Academy of Sciences, Shanghai, 200032, China; National Key Laboratory of Plant Molecular Genetics, CAS Centre for Excellence in Molecular Plant Sciences, Institute of Plant Physiology and Ecology, Chinese Academy of Sciences, Shanghai, 200032, China; Section of Molecular Plant Biology, Department of Biology, University of Oxford, Oxford OX1 3RB, UK; National Key Laboratory of Plant Molecular Genetics, CAS Centre for Excellence in Molecular Plant Sciences, Institute of Plant Physiology and Ecology, Chinese Academy of Sciences, Shanghai, 200032, China

**Dear Editor**,

We welcome this opportunity to give an alternative and balanced perspective on the issues raised in the letter of van Wijk and Adam (2024). At the outset, we wish to make 2 general points that have a significant bearing on the discussion. The first point is that we—the authors of the Li et al. (2022) and Sun et al. (2022) papers—conducted our studies entirely independently, without cooperation or communication between the 2 groups, and yet we reached very similar conclusions. The second point is that we are not dogmatic about the interpretation of our results and the corresponding conclusions. On the contrary, we have been led only by the results we obtained and not by any preconceived ideas of what should or should not be the case. The difficulty with holding a dogmatic position is that it can polarize debate and entrench opinion, ultimately stifling scientific progress. Our view is that this should be avoided. Thus, we are open-minded to the possibility that there are different interpretations of our data (i.e. other than those presented in the Li et al. and Sun et al. reports), provided they are based on objective appraisal of the results. It is important to recognize that an unexpected result is not necessarily an invalid result.

The ubiquitin–proteasome system (UPS) is the preeminent proteolytic system in eukaryotic cells. Its components are abundant in the cytosol and nucleus, and it selectively targets many proteins in both of these compartments for degradation. However, its functions are not restricted to nucleocytosolic proteins as it is now very well established that the UPS also targets proteins in organelles. For example, endoplasmic reticulum (ER) proteins are commonly degraded by the cytosolic proteasome following their extraction (or retrotranslocation) from the organelle, in a process termed ER-associated protein degradation (ERAD). In recent years, it has emerged that even proteins in endosymbiotic organelles (mitochondria and chloroplasts) are processed by ERAD-analogous systems. Ubiquitin-dependent degradation of chloroplast-resident proteins was initially described as a regulatory mechanism governing the chloroplast protein import machinery. This so-called chloroplast-associated protein degradation (CHLORAD) system was assumed to act only at the surface of the organelle owing to the physical barrier presented by the double envelope membrane. However, recent results suggested that the UPS may have a more extensive role in regulating chloroplast proteins, affecting even those located internally. Although somewhat surprising, these results were not entirely unpredictable given that there are numerous historical reports suggesting ubiquitin action in chloroplasts, and there is increasing evidence that the UPS acts on internal mitochondrial proteins.

On reading the letter of van Wijk and Adam, we were initially struck by an apparent incongruity between some of the expressed viewpoints and observations. On the one hand, it is argued that there is not, or cannot be, any ubiquitination in chloroplasts; and then on the other hand, a range of scenarios (including autophagy, stress-related chloroplast damage, and so on) are described in which chloroplast protein ubiquitination might or indeed does occur. These seemingly contradictory positions arise in part because their analysis attempts to make a clear distinction between circumstances in which the chloroplasts are intact, and situations in which organellar integrity is changed or compromised, for example as a result of stress conditions. However, because chloroplasts are highly dynamic organelles that project flexible tubules into the cytosol (so-called stromules) and release a variety of vesicles containing organellar components (Otegui 2018; Hanson and Conklin 2020; Woodson 2022), there may not actually be such a clear distinction in planta. That being said, most of the analyses discussed here were conducted using chloroplasts purified using methods that are broadly accepted to yield intact organelles.

van Wijk and Adam present 3 criteria that they argue must be fulfilled in order for ubiquitin-dependent proteasomal degradation of internal chloroplast-resident proteins to occur. These are as follows: (i) free ubiquitin should be present in chloroplasts; (ii) E1, E2, and E3 enzymes should be present in chloroplasts; and (iii) an export pathway should exist for the delivery of target proteins across both envelope membranes of the organelle. Broadly speaking, we agree with this assessment, albeit with certain caveats, but we do not think it should be necessary to have empirical evidence for all 3 points before concluding that chloroplast proteins may be processed in this way. With regard to the first criterion, some evidence for the presence of free ubiquitin in chloroplasts was previously presented by Wolf et al. (1993). Here, we present new, more robust experimental evidence supporting the existence of free ubiquitin in chloroplasts (Fig. 1); these experiments analyzed either ubiquitin fused to an epitope tag (6×Myc) to improve detection sensitivity and specificity (note that this fusion was not engineered to possess a chloroplast transit peptide) (Fig. 1A) or native ubiquitin (Fig. 1B). The fact that the detected signals were resistant to thermolysin protease treatment of the isolated organelles (which removes externally exposed proteins) argues against the possibility that the relevant proteins were peripherally associated, while the use of multiple antibodies for detection argues against the possibility of insufficient specificity. The relevant chloroplast purification protocol is well established and known to yield minimal contamination (Kubis et al. 2008), and this point was clearly verified using the samples employed here (Fig. 1C). It should also be noted that free ubiquitin is generally observed to be present at low levels, even in nucleocytosolic compartments (Beers et al. 1992; Zhang et al. 2022).

**Figure 1.**
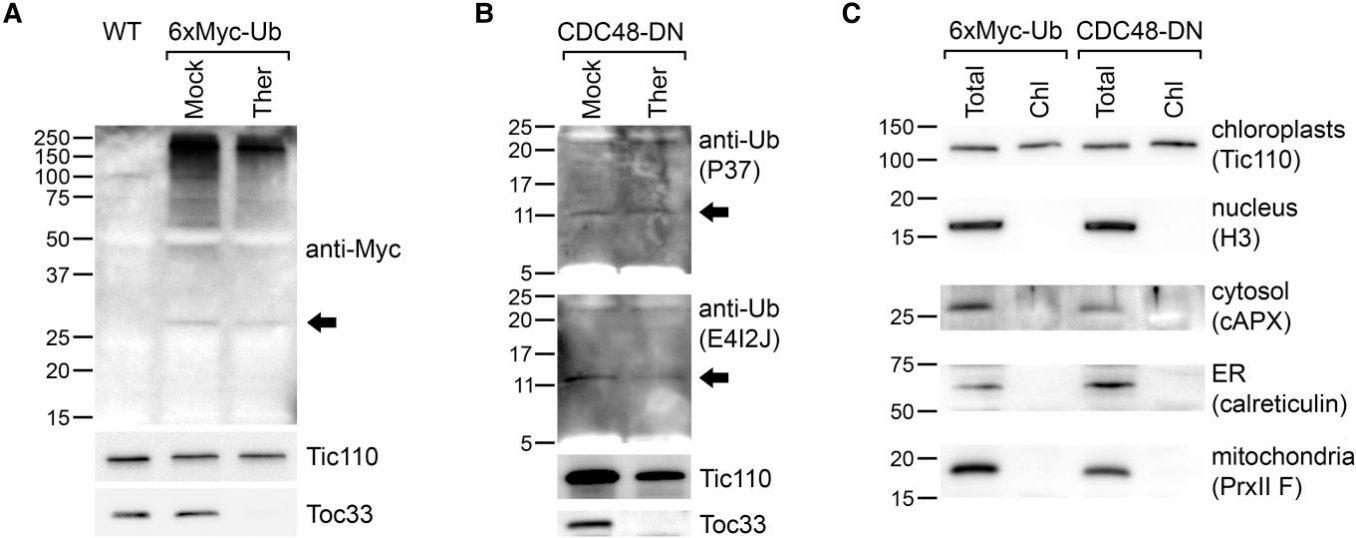
Immunoblotting evidence for the presence of free ubiquitin in isolated chloroplasts. **A)** Chloroplasts isolated from transgenic plants expressing Myc-tagged ubiquitin (6×Myc-Ub) (Sun et al. 2022) were analyzed by immunoblotting using anti-Myc antibody (Cell Signaling Technology, 2276S). The chloroplasts were isolated from 10-d-old seedlings that had been treated with 5 *μ*m bortezomib (Selleckchem, S1013) for the last 2 d. Chloroplasts were treated with 200-*μ*g/mL thermolysin (Ther) protease or buffer lacking protease (Mock), before immunoblotting analysis as described previously (Sun et al. 2022). The arrowhead indicates the lowest molecular weight band that was specifically detected in the transgenic samples, which may be free 6×Myc-Ub protein. The calculated molecular weight of the 6×Myc-Ub protein is 19.07 kDa, and its calculated pI is 4.21 (Expasy); thus, the slow gel migration of the protein is likely due to its acidity. Analysis of control proteins (i.e. Tic110 and Toc33), using antibodies that were previously described (Sun et al. 2022), confirmed the efficacy of the protease treatment. Positions of molecular weight markers (sizes in kDa) are shown to the left of the images. **B)** Chloroplasts isolated from transgenic plants expressing dominant-negative CDC48 (CDC48-DN) (Ling et al. 2019) were analyzed by immunoblotting using different anti-ubiquitin antibodies. The chloroplasts were isolated from 10-d-old seedlings that had been induced with 4 *μ*m estradiol for the last 2 d, as described previously (Ling et al. 2019; Sun et al. 2022). Chloroplasts were treated with 500-*μ*g/mL Ther protease or buffer lacking protease (Mock). Following lysis, the samples were concentrated by ultrafiltration (Sartorius) and analyzed by immunoblotting. Two different anti-ubiquitin antibodies (Cell Signaling Technology, 58395S [P37] and 43124S [E4I2J]) were used to detect ubiquitin, with very similar results. These membranes were cut at the 50-kDa position, and only the lower half was analyzed in each case, to facilitate detection of low molecular weight bands. The arrowheads indicate the lowest molecular weight band that was detected in each case, which may be free ubiquitin. Analysis of control proteins (i.e. Tic110 and Toc33) confirmed the efficacy of the protease treatment. Positions of molecular weight markers (sizes in kDa) are shown to the left of the images. **C)** The chloroplast samples used in **A)** and **B)** (those without Ther treatment) (Chl) were reanalyzed alongside equivalent whole-cell extracts (Total) by immunoblotting, to assess for possible contamination of the chloroplast preparations from other cellular compartments. The blots were probed with antibodies against proteins from different subcellular compartments, as indicated. The chloroplast protein Tic110 served as a chloroplast control in each case. Antibodies against calreticulin and PrxII F were previously described (Finkemeier et al. 2005; Teh and Moore 2007). Antibodies against H3 and cAPX were commercially obtained (H3: Abcam, ab1791; cAPX: Agrisera, AS06 180). Positions of molecular weight markers (sizes in kDa) are shown to the left of the images. cAPX, ascorbate peroxidase, cytosolic; H3, histone H3; PrxII F, Type II peroxiredoxin F.

With regard to the second criterion of van Wijk and Adam, 2 points are highly relevant. First, it is conceivable that any ubiquitination machinery present inside chloroplasts is noncanonical in character, perhaps especially so given the prokaryotic origin of the organelle (Berk and Hochstrasser 2016; Qiu et al. 2016; Ledvina et al. 2023). Indeed, the established CHLORAD E3 ligase, SUPPRESSOR of *PPI1* LOCUS1 (SP1), is not implicated in the ubiquitination of internal chloroplast proteins (Sun et al. 2022). Second, while it is true that the recognizable E1–E2–E3 components do not have recognizable chloroplast transit peptides, this is not strong evidence that ubiquitination machinery is absent from chloroplasts. Indeed, it is well documented that transit peptide prediction methods are imperfect (Emanuelsson et al. 2007) and, furthermore, that a significant number of chloroplast proteins (as many as 30% according to one estimation) gain access to the organelle via nonstandard pathways that do not require a transit peptide (Kleffmann et al. 2004; Nada and Soll 2004; Miras et al. 2007; Armbruster et al. 2009). Moreover, the assertion that there is no evidence in the SUBcellular location database for *Arabidopsis* proteins (SUBA) for the chloroplast localization of E1, E2, or E3 enzymes is incorrect. On the contrary, a number of such proteins (>10) are annotated as being potentially chloroplast-localized based on MS or microscopy data (Hooper et al. 2017), although further biochemical and functional analyses will be required to verify the annotations in most cases. It is true that in earlier work, such proteins were not identified by proteomics, although this was possibly due to sensitivity limitations of MS technology at the time; even now, E3 ligases are prominent constituents of the “dark proteome” that has evaded proteomic detection (van Wijk et al. 2023). In support of this view, although SP1 was identified genetically as a chloroplast E3 in 2012, it was not identified by proteomics until 2019 (Ling et al. 2012; Bouchnak et al. 2019); and we now routinely identify SP1 in purified chloroplasts in our own MS analyses. It is also noteworthy that subcellular localization analysis by confocal microscopy identified another biochemically active E3, SHOOT GRAVITROPISM9, as being likely localized inside plastids (Nakamura et al. 2011). This protein controls gravity sensing, and it will be intriguing to determine what its substrates are in the future.

The third criterion related to the need for a protein export pathway is indeed a significant challenge for future research. However, given that there are many proteins in the chloroplast envelope of unknown function (Bouchnak et al. 2019), there is no clear reason to suppose that such machinery is absent. Indeed, the CELL DIVISION CYCLE48 (CDC48)-dependence of the processing of ubiquitinated chloroplast proteins (Li et al. 2022; Sun et al. 2022) does point to the existence of an export system.

The possibility that ubiquitination has a significant role to play in chloroplasts has been considered many times previously, and van Wijk and Adam included in their letter an appraisal of some of the relevant literature. However, we do not find this appraisal to be well balanced, nor do we accept the implied conclusion that all of the historical studies pointing toward a role for ubiquitination in chloroplasts were fundamentally flawed. For example, Wettern et al. (1990) presented clear anti-ubiquitin immunogold signals in *Chlamydomonas* that were quite localized in certain organelles, including the chloroplast, vacuole, and nucleus; we do not agree with the assertion that the particles were nonspecifically distributed and hard to recognize. Veierskov and Ferguson (1991) provided evidence for the ubiquitin-dependent breakdown of oat Rubisco using ^125^I protein labeling, an observation that aligns intriguingly with the finding by Li et al. (2022) and Sun et al. (2022) that rbcL is a significant ubiquitination target. Hoffman et al. (1991) analyzed fractions of purified spinach chloroplasts by immunoblotting and detected ubiquitin conjugates in both thylakoidal and stromal fractions. Importantly, the isolated chloroplasts were treated with thermolysin before fractionation, suggesting that the detected ubiquitinated proteins were not merely associated with the chloroplast surface. Beers et al. (1992) detected very weak signals (smears) in *Arabidopsis* chloroplast stromal fractions upon anti-ubiquitin immunoblotting. While the authors attributed these weak signals to cytosolic contamination (based on superficial between-fraction similarities in the smeared signals), an alternative interpretation that these were true chloroplast protein signals cannot be ruled out given that cytosolic marker enzyme activity was absent from the stromal fraction. However, this study was largely inconclusive owing to the weakness of the detected smears even in whole-leaf extracts. Ubiquitination levels in chloroplasts would be expected to be much lower than those in whole-leaf extracts and, in our experience, only become prominent when CDC48 activity is impaired (Sun et al. 2022). As noted earlier, Wolf et al. (1993) detected what appeared to be free ubiquitin in *Vicia faba* chloroplasts by immunoblotting. While the Ramos et al. (1995) study highlighted by van Wijk and Adam did indeed reach opposing conclusions on chloroplast protein ubiquitination, it should be noted that this work was also hampered by weak signal intensities even in the positive controls (making it hard to draw convincing conclusions about the chloroplasts) and that it has not been well cited. Thus, it is difficult to sustain an argument that this study is somehow the basis for a consensus view or dogma in the field that chloroplasts lack ubiquitination.

van Wijk and Adam are also critical of the results presented more recently by Li et al. (2022) and Sun et al. (2022). In particular, it is argued that the detection of polyubiquitinated proteins in isolated chloroplasts by Li et al. (2022) was not persuasive due to the absence of a postisolation protease treatment step (even though the analysis of a soluble stromal fraction arguably already excluded the possibility that the ubiquitination was envelope associated) and that the similar detection of such proteins by Sun et al. (2022) would have been more persuasive had an anti-ubiquitin antibody been used. Here, we present experiments that directly address both of these criticisms. These new data clearly show that polyubiquitinated proteins are present in isolated chloroplasts even after protease treatment and regardless of which antibody is used for detection (Fig. 2). Despite raising doubts about the reliability of the evidence for the presence of ubiquitination in chloroplasts, as noted above, van Wijk and Adam go on to infer, in any case, that such ubiquitination might have been detected due to the use of organelles with compromised integrity (related to, for example, stressful growth conditions). In this regard, we wish to emphasize that in the relevant experiments of both Li et al. (2022) (e.g. figure 1) and Sun et al. (2022) (e.g. figures 1 and 2), stressful conditions were not employed. Moreover, we here present empirical evidence that the organelles in CDC48-DN plants (which were used extensively in the study of Sun et al. 2022) are intact (Fig. 3). Lastly, van Wijk and Adam are critical of the ubiquitinomic analysis presented by Sun et al. (2022), particularly in relation to the use of alkylating agents. This concern is based on knowledge that the use of iodoacetamide (IAA) can sometimes present problems in ubiquitinomic studies (Nielsen et al. 2008). However, it should be noted that IAA was not used in any of the ubiquitinomic analyses of Sun et al. (2022); instead, the widely accepted alternative reagent, chloroacetamide (CAA) (Nielsen et al. 2008), was employed in these experiments. Thus, we do not accept that the resulting conclusions were unreliable.

**Figure 2.**
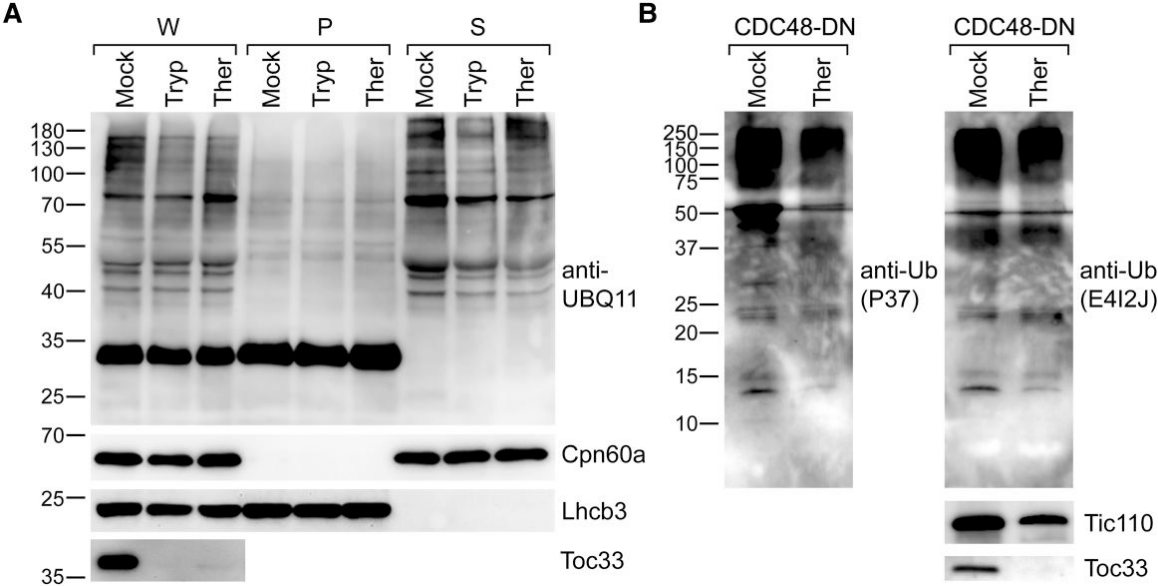
Immunoblotting analyses showing the presence of ubiquitination in isolated chloroplasts following protease treatment and using different antibodies. **A)** Detection of ubiquitinated proteins in chloroplasts isolated from wild-type plants. The chloroplasts were isolated from 10-d-old seedlings as described previously (Li et al. 2022). Chloroplasts were treated with 100-*μ*g/mL trypsin (Tryp) or thermolysin (Ther) protease or buffer lacking protease (Mock), before lysis, according to Froehlich (2011). Proteins in the whole chloroplast (W), pellet (P), and supernatant (S) subfractions (Li et al. 2022) were analyzed by immunoblotting. An anti-UBQ11 antibody (Agrisera, AS08307A) was used to detect ubiquitination. Analysis with anti-Cpn60a or anti-Lhcb3 antibodies (Li et al. 2022) provided controls for successful fractionation and equal loading, while analysis with anti-Toc33 confirmed the efficacy of the protease treatments. Positions of molecular weight markers (sizes in kDa) are shown to the left of the images. **B)** Detection of ubiquitinated proteins in chloroplasts isolated from CDC48-DN plants. The chloroplasts were isolated from 10-d-old CDC48-DN transgenic seedlings that had been induced with 4-*μ*m estradiol and were treated with 500-*μ*g/mL Ther protease or buffer lacking protease (Mock), before immunoblotting analysis (the samples are the same as those used in Fig. 1, B and C). Four different anti-ubiquitin antibodies (Cell Signaling Technology, 58395S [P37]; Cell Signaling Technology, 43124S [E4I2J]; Agrisera, AS08307; Invitrogen, 14-6078-82) were used to detect ubiquitination of chloroplast proteins, and similar results were observed. The results obtained with two of these antibodies (P37 and E4I2J) are shown here. These membranes were cut at the 50-kDa position, and the slices were incubated separately with the same antibody at the same dilution to facilitate detection of low molecular weight bands. Analysis of control proteins (i.e. Tic110 and Toc33) confirmed the efficacy of the protease treatment. Positions of molecular weight markers (sizes in kDa) are shown to the left of the images.

**Figure 3.**
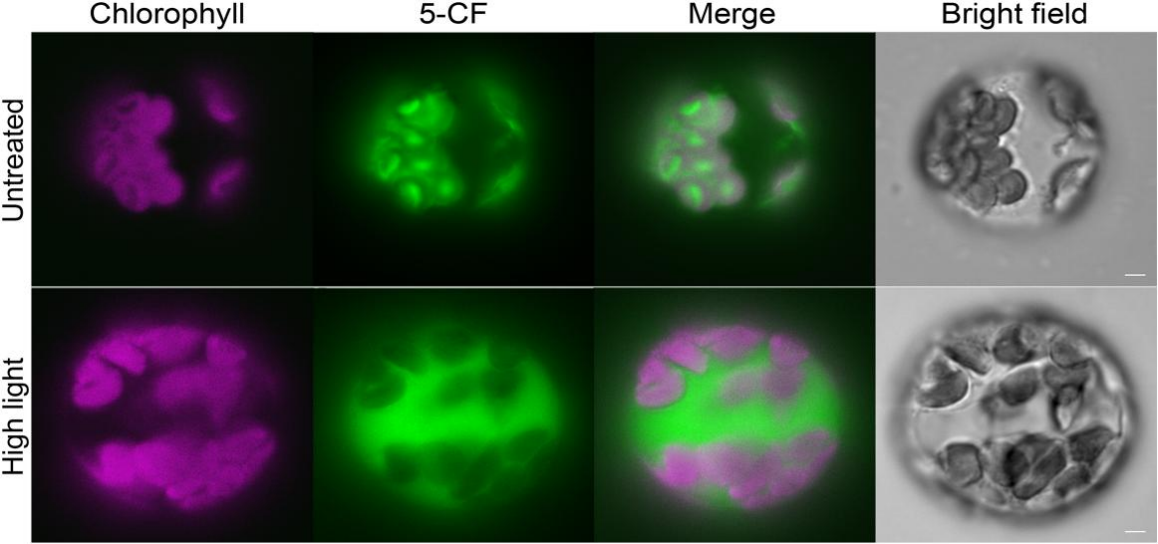
Assay of chloroplast membrane integrity in plants expressing the dominant-negative mutant of CDC48 (CDC48-DN). Protoplasts were isolated from 8-d-old CDC48-DN seedlings, following a 2-d estradiol induction, as previously described (Ling et al. 2019), and were then incubated with 5-mg/L carboxyfluorescein diacetate (CFDA) for 5 min at room temperature in the dark. CFDA is converted to fluorescent 5-carboxyfluorescein (5-CF) in the chloroplast upon hydrolysis by stromal carboxylases (Schulz et al. 2004). Thus, if the chloroplast membranes are broken or have been broken and resealed, these carboxylases are lost from the stroma resulting in chloroplasts that lack 5-CF fluorescence. For the positive control (high light stress), the protoplasts were treated with 500-*µ*mol/m^2^/s actinic light for 15 min, after isolation but before incubation with CFDA, in order to damage chloroplast membrane integrity. The 5-CF fluorescence and chlorophyll autofluorescence signals were visualized by microscopy under epifluorescence using a YFP filter cube (excitation 513 nm; emission 548 nm) and a chlorophyll filter cube (excitation 438 nm; emission 679 nm), respectively. Scale bars represent 5 *µ*m.

We apologize for the confusion regarding the use of different alkylating agents in the Sun et al. (2022) study, since it appears that the descriptions we provided in that paper were not sufficiently clear. While IAA was used in the separate quantitative proteomic analyses, this reagent was not used in the ubiquitinomic analyses (where excessive alkylation could potentially be problematic). We used CAA in all of the ubiquitinomic analyses—a point that was made clearly in the relevant database accessions, although admittedly not in the paper itself. We are of course aware of the problems that the use of IAA in such analyses can potentially present (Nielsen et al. 2008), although we also note that this is not always the case (Bustos et al. 2012; Anania et al. 2014; Maxwell et al. 2021). Even though IAA was not used in our ubiquitinomics, it is worth mentioning that IAA-induced lysine alkylation at room temperature is in any case a rather rare side reaction in the preparation of MS samples (Suttapitugsakul et al. 2017).

It is entirely relevant and appropriate to consider what is known about ubiquitination in the other endosymbiotically derived organelle—the mitochondrion—when discussing the evidence for ubiquitination in chloroplasts. But, of course, such analysis is only useful if it is done with objectivity and balance. In fact, there is accumulating evidence that ubiquitination affects internal mitochondrial proteins. Immunoelectron microscopy evidence for the localization of an E1 protein inside human mitochondria was presented decades ago (Schwartz et al. 1992). More recently, a number of studies pointed to internal mitochondrial proteins (including inner membrane and matrix proteins) being targets of the UPS (Margineantu et al. 2007; Azzu and Brand 2010; Clarke et al. 2012; Lehmann et al. 2016; Lavie et al. 2018; Liao et al. 2020). One recent study by Zhang et al. (2022) systematically demonstrated that several UPS components, including E1, E2, and E3 enzymes, are localized in the yeast mitochondrial matrix using a genetic α-complementation assay. This study also presented biochemical evidence that ubiquitination occurs inside mitochondria and that the Rad6 E2 affects the pattern of mitochondrial protein ubiquitination. We do not agree with the claim by van Wijk and Adam that such findings are ignored in the mitochondrial literature. On the contrary, UPS-mediated control of mitochondrial internal proteins is discussed in both of the reviews that were cited in support of this claim (Rödl and Herrmann 2023; Uoselis et al. 2023), with one of them specifically commenting that the underlying mechanisms will be valuable to decipher.

The further argument of van Wijk and Adam that organelles possessing an abundant internal Clp protease system (like chloroplasts) might exclude other, external protein degradation systems is not persuasive in our view. It is well known that mitochondrial protein homeostasis requires both internal degradation by proteases and external degradation via the UPS or autophagy, with a retrotranslocation or extraction step being a prerequisite for the latter (Uoselis et al. 2023). Indeed, it is quite common that an organelle or even an individual substrate protein can be degraded by multiple proteolytic pathways (Nolan et al. 2017; Wang et al. 2021; Costigliolo Rojas et al. 2022; Park et al. 2022). Many proteins of the ER are subject to degradation by the cytosolic proteasome, following their ubiquitination and extraction through CDC48 (even ER lumen proteins are ubiquitinated, after their initial dislocation to the ER surface). This process, termed ERAD, is a dominant mechanism of protein homeostasis for this organelle (Christianson et al. 2023). In spite of the importance of the ERAD system, ER proteins are also frequently processed by autophagy (Clavel and Dagdas 2021; Sun et al. 2021).

We wish to conclude by countering the “take-home” message of van Wijk and Adam with some of our own, which we believe are more reflective of all of the available data. First, we find that the evidence for the existence of polyubiquitination inside chloroplasts (both from our own work and from many previous studies) is strong. Of course, it is possible to identify weaknesses in any one individual experiment, but when all of the evidence is considered together in the round, it becomes persuasive in our view; one can question how the ubiquitination gets there, and why it is there, but not easily that it exists at all. Second, while we accept that there are many significant (and highly interesting) outstanding questions pertaining to how internal chloroplast protein ubiquitination is delivered and processed, we contend that there are no convincing arguments that preclude the existence of ubiquitination machinery, of some sort, inside the organelle, or of export machinery in the envelope membranes. An absence of evidence does not equate to evidence of absence. Thus, we look forward to seeing these exciting questions being addressed by the field in the coming years.

## Data Availability

The data underlying this article are available in the article.

## Dive Curated Terms

The following phenotypic, genotypic, and functional terms are of significance to the work described in this paper:

PPI1 Gramene: AT1G02280
PPI1 Araport: AT1G02280

proteins CHEBI: CHEBI:36080

SHOOT GRAVITROPISM9 Gramene: AT5G02750
SHOOT GRAVITROPISM9 Araport: AT5G02750

SUPPRESSOR OF PPI1 LOCUS1 Gramene: AT1G63900
SUPPRESSOR OF PPI1 LOCUS1 Araport: AT1G63900
